# Effectiveness of Educational Videos in Encouraging Preferences for Guideline-Based Cancer Screening in Japan: Three-Arm Pseudorandomized Controlled Trial

**DOI:** 10.2196/82322

**Published:** 2026-02-12

**Authors:** Kosuke Sakai, Yuko Furuya, Shoko Nakazawa, Kota Fukai, Kei Sano, Masayuki Tatemichi

**Affiliations:** 1 Department of Preventive Medicine Tokai University, School of Medicine Kanagawa Japan; 2 Department of Ophthalmology The Jikei University, School of Medicine Tokyo Japan

**Keywords:** health education, randomized controlled trial, cancer screening, false positive, decision-making

## Abstract

**Background:**

Although cancer screening is essential for early detection and an improved prognosis, screening beyond the recommended guidelines may increase the risk of false-positive results. Consequently, educating individuals about the potential harm of non–guideline-based cancer screening is essential; however, effective communication methods remain unclear.

**Objective:**

This study aimed to evaluate the effectiveness of different types of educational videos in encouraging preferences for guideline-based cancer screening.

**Methods:**

This 3-arm pseudorandomized controlled trial was conducted in June 2025 using a Japanese online survey platform. Eligible respondents were working adults aged 30 to 60 years with no history of major cancer. Respondents were assigned to 1 of the following 3 video conditions: video A, which provided a logical explanation of false-positive risks; video B, which presented the narrative of a woman who received a false-positive result from breast cancer screening; and video C, which depicted a man who underwent unnecessary follow-up testing after tumor marker screening. The primary outcome was the preference for guideline-based cancer screening after watching the videos. The secondary outcomes included 7 self-reported video evaluation items, such as perceived relevance and clarity, assessed using a 5-point Likert scale. Differences in the primary outcome between video groups were analyzed using multivariable logistic regression with adjustment for covariates. Means and 95% CIs were calculated for each secondary outcome according to sex and video group. In addition, before-and-after changes in screening preferences were assessed using McNemar test, with a significance level of .05.

**Results:**

In total, 1200 respondents (400 per group) completed the survey. No statistically significant differences in the primary outcome were observed among the video groups. With reference to video A, the adjusted odds ratios for preferring guideline-based screening were 0.89 (95% CI 0.59-1.32) for video B and 0.98 (95% CI 0.65-1.46) for video C. Regarding secondary outcomes, male respondents rated video B less favorably than female respondents in terms of relevance and willingness to undergo guideline-based screening. The before-and-after comparison showed a significant change in preference for guideline-based screening (*P*=.04). These videos appeared to be more effective for individuals with an annual history of colorectal cancer screening than for those without such a history.

**Conclusions:**

Educational videos have the potential to influence cancer screening preferences; however, no single video format has demonstrated clear superiority. These findings underscore the importance of tailoring educational materials to the target audience characteristics. Further research is required to develop effective strategies for encouraging guideline-based cancer screening.

**Trial Registration:**

University Hospital Medical Information Network Clinical Trials Registry UMIN000060549; https://center6.umin.ac.jp/cgi-open-bin/ctr/ctr_view.cgi?recptno=R000066119

## Introduction

The strategic implementation of cancer screening facilitates early cancer detection, thereby improving patient survival through less invasive treatment options [1]. National guidelines, such as those developed by the US Preventive Services Task Force and the Japan National Cancer Center, clearly define the populations that benefit from screening and the tests recommended for them, based on robust evidence of mortality reduction [2,3]. Despite these guidelines, many individuals still undergo nonrecommended screenings, including breast cancer screening in people younger than the recommended age and whole-body positron emission tomography-computed tomography (PET-CT) imaging [4,5].

These non–guideline-based screenings often cause harm to participants through false-positive results, overdiagnosis, and overtreatment [6]. False-positive results often lead to negative psychosocial consequences [7]. In addition, prostate, lung, colorectal, and ovarian cancer screening trials reported that undergoing up to 14 cancer screening tests over a 3-year period resulted in cumulative risks of 60.4% and 48.8% for experiencing at least 1 false-positive result in men and women, respectively [8]. Moreover, approximately 10% of Japanese women in their 40s have been reported to experience false-positive results during breast cancer screening [9]. This risk is considered further elevated when screening is conducted at a younger age [10]. Furthermore, previous studies [11,12] have suggested that false-positive results can result in decreased adherence to future cancer screening. Accordingly, health care professionals play a crucial role in facilitating informed decision-making in cancer screening by helping participants understand both the benefits and harms of screening [13,14].

Previous studies [14,15] have evaluated the effects of various educational programs aimed at enhancing knowledge of the benefits and harms of cancer screening. For instance, multicomponent interventions based on behavior change models were implemented at the community level for breast cancer education [15]. These interventions were reported to be successful when various factors were considered, including the use of video consultations, provision of sessions in multiple languages, and consideration of the socioeconomic status of the community [16]. However, these educational efforts were designed to increase cancer screening uptake and not to alert people to the harmful effects of non–guideline-based cancer screening.

In Japan, the commercialization of health care has led to a strong emphasis on the benefits of early cancer detection and additional testing as much as possible. A survey on the implementation status of cancer screening in Japan [17] found that approximately 70% of companies offered colorectal cancer screening to individuals younger than 40 years, and 45% offered breast cancer screening to individuals younger than 40 years. A national survey in Japan [18] has shown that more than 50,000 healthy individuals in 2005 and approximately 39,000 per year from 2006 to 2009 underwent PET-CT cancer screening outside of guideline-based programs. Therefore, educating individuals about the potential harms of non–guideline-based cancer screening is essential for informed decision-making; however, effective communication methods remain unclear. One educational method can be to raise awareness about the risks associated with false-positive results.

We aimed to compare the short-term impact of 3 brief educational videos on preferences for guideline-based cancer screening. This intervention was grounded in the narrative transportation theory, which suggests that stories transport audiences into a narrative world, focusing attention, emotion, and imagery on the story and thereby reducing counterarguing and shaping beliefs and attitudes [19]. In this framework, we hypothesized that narrative videos would be more effective than an explanatory format in shifting preferences toward guideline-based screening. By comparing the effectiveness of the 3 types of educational videos, we gained deeper insight into how approaches are most effective for specific characteristics of the target audience.

## Methods

### Study Design and Respondents

This study was conducted according to the protocol registered in the University Hospital Medical Information Network Clinical Trials Registry (UMIN000060549). In June 2025, we conducted a 3-arm pseudorandomized controlled trial using a Japanese online survey platform. This trial was supported by Rakuten Insight Inc, a web-based research company located in Tokyo. The respondents were recruited from the company’s registry via email. This manuscript is reported in accordance with the Consolidated Standards of Reporting Trials (CONSORT) 2010 statement (Multimedia Appendix 1).

The inclusion criteria were working individuals aged between 30 and 60 years who agreed to participate in the study. The exclusion criteria were a history of stomach, lung, colon, breast, or cervical cancer. In addition, individuals who reported undergoing cancer screening recommended by local governments within the past 3 years were excluded from the study, as these programs only offer screening options that adhere to national guidelines. The participants were administered a questionnaire comprising 27 questions. Respondents who required <5 minutes or >30 minutes to complete the questionnaire were considered to have given inappropriate answers and were excluded.

### Randomization and Procedure

The respondents were not individually randomized. Instead, separate web-based questionnaires were prepared for each of the 3 video conditions (A, B, and C). Invitations were sent to participants until 400 valid responses were obtained for each video group. Invitations for each group were distributed sequentially and exclusively, and individuals who were invited to one video condition were not invited to any other condition. The target population included 200 men and 200 women in 1 group, resulting in 3 groups with a total target sample size of 1200 respondents. Each group viewed a different video. We ensured that the respondents were unable to answer the next question unless they watched the video until the end, to ensure data quality. The sample size was determined based on previous studies of educational videos using animation [20].

### Intervention

We developed 3 educational videos to inform people about the potential harm caused by false-positive results in cancer screening and to encourage guideline-based screening decisions. Each educational video was developed based on the health belief model [21], which explained that health-related behavioral change can be influenced by individuals’ perceptions of expected benefits and costs. In the context of cancer screening, the perceived benefits of guideline-based screening were detecting cancer at an earlier stage, enabling less invasive treatment, and potentially reducing cancer mortality. The costs included financial and time burdens, psychological distress triggered by false-positive results, and potential harm from unnecessary follow-up procedures such as avoidable radiation exposure. By presenting the benefits of guideline-concordant screening and the potential harms of non–guideline-based screening, the videos were designed to support informed decision-making and encourage preferences aligned with screening guidelines.

All videos had a duration of approximately 100 seconds. Video A illustrated the disadvantages of excessive cancer screening using charts and numerical data. Video B showed the story of a woman in her 30s who underwent breast cancer screening, was referred for further testing, and ultimately received a false-positive result. Video C showed the story of a working man who underwent multiple tumor marker tests, received positive results in several categories, and was subsequently required to undergo numerous further tests. The structures of the 3 videos are shown in Multimedia Appendix 2. Video A is shown in Multimedia Appendix 3; video B is shown in Multimedia Appendix 4; and video C is shown in Multimedia Appendix 5. The duration of video A was 1 minute and 24 seconds, whereas videos B and C lasted for 1 minute and 17 seconds each.

### Outcome Measures

The primary outcome was preference for guideline-based cancer screening after watching the videos. Respondents were asked what type of cancer screening they would prefer, with the following options: (1) “I want to undergo cancer screening as recommended by the guidelines,” (2) “I want to undergo cancer screening at an earlier age than recommended,” (3) “I want to undergo different types of cancer screening than recommended (eg, tumor markers or PET-CT imaging),” (4) “I want to undergo cancer screening at intervals shorter or longer than the recommended interval,” (5) “I do not want to undergo any cancer screening,” and (6) “I have no idea.” Options 2, 3, and 4 were not mutually exclusive, and if any of these options were chosen, the response was interpreted as a preference for non–guideline-based cancer screening. The primary outcome measured an immediate postexposure preference and was intended as a proximal indicator of decision-making.

We included a 7-item evaluation survey as a brief process evaluation to assess how participants received the educational messages and to aid interpretation of the primary outcome. Conceptually, the items included (1) perceived personal relevance and engagement with the message (relevance), which is central to narrative persuasion and transportation; (2) comprehensibility and cognitive processing of the content (clarity), which is a prerequisite for informed decision-making; (3) perceived informational sufficiency for making a screening decision (informativeness), corresponding to the “knowledge or understanding” component needed to weigh benefits and harms; (4) perceived acceptability as educational material (acceptability), reflecting practical feasibility and appropriateness for dissemination; and (5) negative affect and avoidance tendency (aversion), which relates to potential unintended emotional burden and message avoidance [22]. Respondents rated these aspects on a 5-point Likert scale (1=strongly disagree, 2=disagree, 3=neutral, 4=agree, and 5=strongly agree): relevance (“This video is relevant to me”), clarity (“This video is clear and easy to understand”), informativeness (“This video provides all the necessary information for screening participants”), acceptability (“This video is acceptable as educational material”), and aversion (“I do not want to think about this video”). In addition, respondents were asked about the influence of the videos on 2 aspects: motivating them to discuss their content with family or friends and undergoing guideline-based cancer screening using the same 5-point scale.

### Covariates

Covariates included sex, age, education, and preferences for cancer screening before viewing the video. Sex was based on self-reported information, and age was determined from the date of birth. Education was categorized into middle school, high school, vocational schools, junior college or technical college, university, graduate school, currently enrolled, and other, and respondents were asked to select the most appropriate option. However, for analysis, education was treated as a binary variable: less than a university degree vs a university degree or higher. The preferences for cancer screening before viewing the video were obtained using the same method as that used for the primary outcome.

### Statistical Analysis

The distributions of age, marital status, education, income, and preferences for cancer screening before watching the video were tabulated for the 3 groups to compare the characteristics of the respondents. The primary outcome variable was coded as 1 if the respondent selected the option “I want to undergo guideline-based cancer screening” after watching the video, and 0 otherwise. The adjusted odds ratios (aORs) and 95% CIs for videos B and C, relative to video A, were estimated using multivariate logistic regression analysis. The covariates included sex, age, education, and a preference for cancer screening before watching the video.

The means and 95% CIs were calculated for each of the secondary outcomes, which were the 7 self-reported video evaluation indicators, stratified by sex. In addition to the prespecified primary between-group comparison, we conducted an exploratory, post hoc before-and-after analysis of screening preferences immediately before and after video viewing across all participants. This analysis was not prespecified in the trial protocol and should be interpreted as descriptive and hypothesis generating. A 2-sided McNemar test was performed at a significance level of .05. Changes in cancer screening preferences before and after viewing the videos were visualized using a Sankey diagram.

We compared 3 groups to examine the characteristics of participants who perceived the video as effective: individuals who shifted toward guideline-based screening (effective group), those whose preferences did not change (neutral group), and those who shifted away from guideline-based screening (counterproductive group). To compare participant characteristics across the 3 groups, *P* values were calculated using Welch 1-way ANOVA for continuous variables and the chi-square test for categorical variables, with a 2-sided significance level set at 5%. These *P* values were provided for descriptive purposes; no adjustment for multiple comparisons was applied. Age and total response time were treated as continuous variables, while age group (10-year categories), sex, marital status, education, income, and history of colorectal cancer screening were treated as categorical variables. All statistical analyses were performed using R (version 4.4.2; R Foundation for Statistical Computing). Because all questions were mandatory, the dataset contained no missing values.

### Ethical Contributions

This study was approved by the Research Ethics Committee of Tokai University School of Medicine (24R099). Informed consent was obtained from all respondents before they responded to the questionnaire. Because cancer-related educational materials may unintentionally elicit fear or distress, potential adverse emotional effects could not be fully excluded. During video production, we intentionally avoided sensational expressions. The video materials were submitted for institutional ethics review and were evaluated from a third-party perspective. Before participation, respondents were informed that the survey included videos addressing the potential harms and benefits of cancer screening. Respondents were also informed that participation was voluntary, that they could discontinue the survey at any time, and that discontinuation would not result in any disadvantage. Participation was incentivized by compensating respondents who completed the survey with points that could be exchanged for merchandise on online platforms. To protect respondents' privacy, we avoided collecting personally identifiable information. We confirmed that the data contained no personal information before conducting our analysis.

## Results

### Characteristics of Respondents

Table 1 summarizes the characteristics of the overall cohort and the individual video groups. The mean age of 1200 participants was 48.9 (SD 7.4) years, and the most common age group was 50 to 60 years (n=647, 53.9%). The proportion of unmarried individuals was 43.4% (n=521), and the most common educational attainment was a university degree (n=502, 41.8%). The most frequently reported income level was ≤JP ¥4 million (US $1.00=JP ¥145 is used throughout the text), with 310 (25.8%) respondents. The most common preference for cancer screening before watching the video was guideline-based screening (n=565, 47.1%).

**Table 1:** Characteristics of all respondents (N=1200).

Characteristics		All videos (n=1200)	Video A (n=400)	Video B (n=400)	Video C (n=400)
Age (years), mean (SD)		48.9 (7.4)	49.13 (7.5)	48.31 (7.8)	49.17 (7.3)
**Age (years), n (%)**					
	30-39	162 (13.5)	48 (12.0)	65 (16.2)	49 (12.2)
	40-49	391 (32.6)	131 (32.8)	133 (33.2)	127 (31.8)
	50-60	647 (53.9)	221 (55.2)	202 (50.5)	224 (56.0)
**Marital status, n (%)**					
	Unmarried	521 (43.4)	180 (45.0)	173 (43.2)	168 (42.0)
**Education, n (%)**					
	Junior high school	9 (0.8)	3 (0.8)	2 (0.5)	4 (1.0)
	High school	299 (24.9)	110 (27.5)	101 (25.2)	88 (22.0)
	Vocational school graduate	160 (13.3)	53 (13.2)	54 (13.5)	53 (13.2)
	Junior college or technical college graduate	139 (11.6)	45 (11.2)	46 (11.5)	48 (12.0)
	University graduate	502 (41.8)	161 (40.2)	166 (41.5)	175 (43.8)
	Graduate school graduate	82 (6.8)	25 (6.2)	29 (7.2)	28 (7.0)
	Currently enrolled	9 (0.8)	3 (0.8)	2 (0.5)	4 (1.0)
**Income (Japanese yen; million** **,** **US $1.00 = ¥145** **), n (%)**					
	≤4.00	310 (25.8)	115 (28.7)	99 (24.8)	96 (24.0)
	4.01-6.00	270 (22.5)	90 (22.5)	100 (25.0)	80 (20.0)
	6.01-8.00	240 (20.0)	80 (20.0)	71 (17.8)	89 (22.2)
	8.01-10.00	163 (13.6)	48 (12.0)	58 (14.5)	57 (14.2)
	10.01-12.00	98 (8.2)	24 (6.0)	32 (8.0)	42 (10.5)
	12.01-15.00	70 (5.8)	27 (6.8)	26 (6.5)	17 (4.2)
	≥15.01	49 (4.1)	16 (4.0)	14 (3.5)	19 (4.8)
**Preference for cancer screening before watching the video, n (%)**					
	“I want to undergo cancer screening in accordance with the guidelines.”	565 (47.1)	177 (44.2)	183 (45.8)	205 (51.2)
	“I want to undergo cancer screening that differs from the guidelines.”	383 (31.9)	135 (33.8)	136 (34.0)	112 (28.0)
	“I don’t want to get screened for cancer.”	67 (5.6)	18 (4.5)	25 (6.2)	24 (6.0)
	“I have no idea.”	185 (15.4)	70 (17.5)	56 (14.0)	59 (14.8)

### Effect of Video Viewing on Cancer Screening Preferences Based on Guidelines

**Figure 1:**
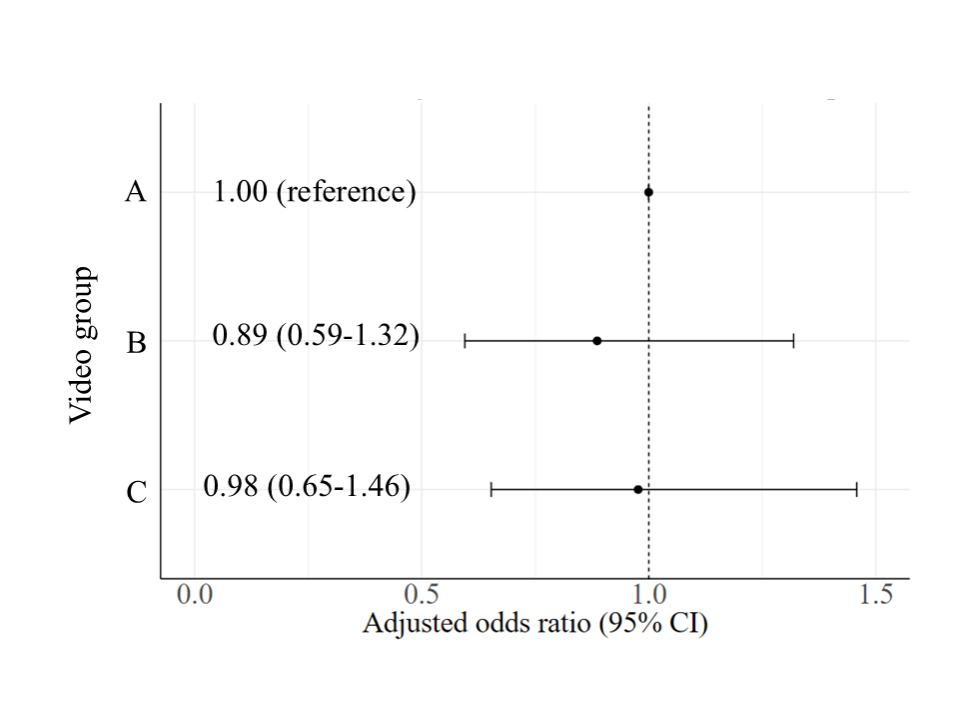
Effect of videos on guideline-based screening preference. Analyses were adjusted for sex, age, education (university degree or lower), and preference for cancer screening before watching the video.

Figure 1 presents the aORs and 95% CIs for the primary outcome based on logistic regression analysis. Compared with video A, the aOR for video B was 0.89 (95% CI 0.59-1.32) and that for video C was 0.98 (95% CI 0.65-1.46), indicating no statistically significant differences. Multimedia Appendix 6 presents the results for all variables included in the logistic regression analysis.

### Evaluation of the Videos

**Figure 2:**
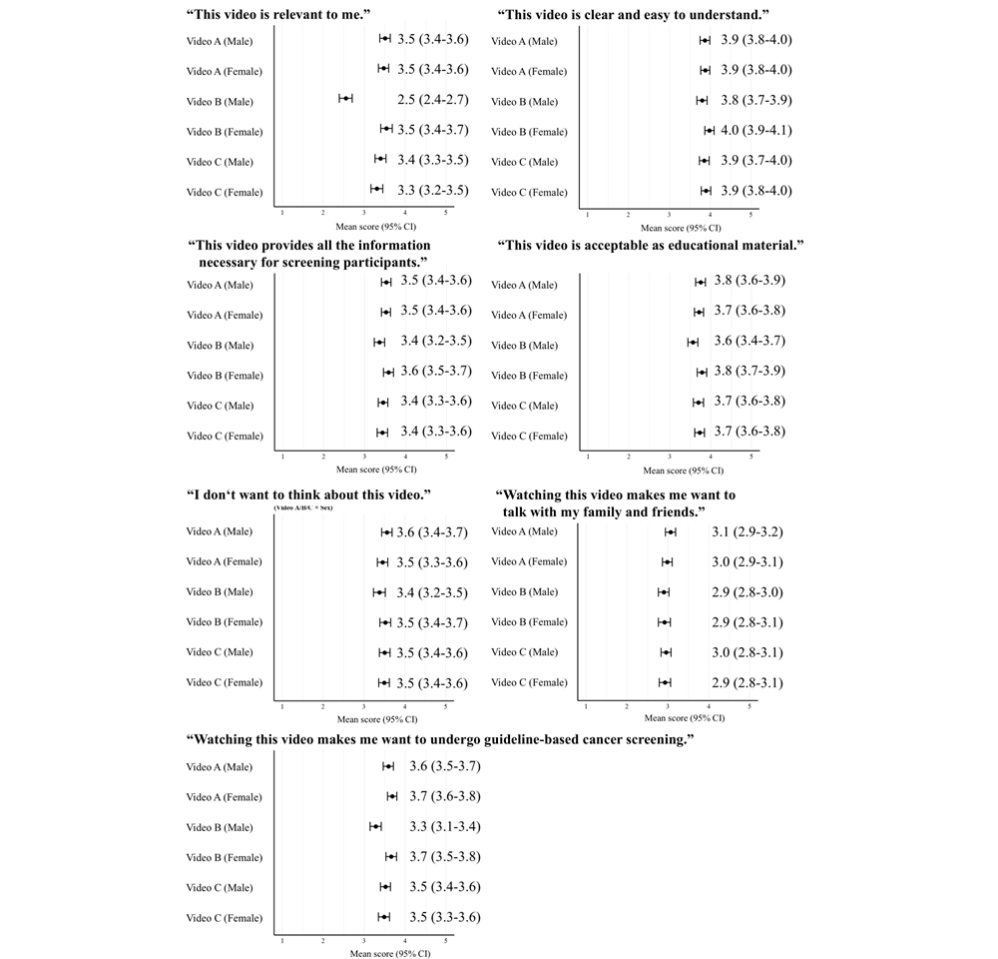
Mean score with 95% CI for each video and sex group.

Figure 2 shows the results of the secondary outcomes (7-item evaluation) for each video stratified by sex. The perceived relevance of video B was significantly lower among men (mean 2.5; 95% CI 2.4-2.7) than among women (mean 3.5; 95% CI 3.4-3.7). Similarly, willingness to undergo guideline-based screening was significantly lower among men (mean 3.3; 95% CI 3.1-3.4) than among women (mean 3.7; 95% CI 3.5-3.8). All scores, rounded to the second decimal place, are presented in Multimedia Appendix 7. Although no statistically significant differences were observed, the point estimates for all evaluation items among the women tended to be equal to or higher for video B than for video C.

### Total Changes in Cancer Screening Preferences Before and After Viewing the Video

**Figure 3:**
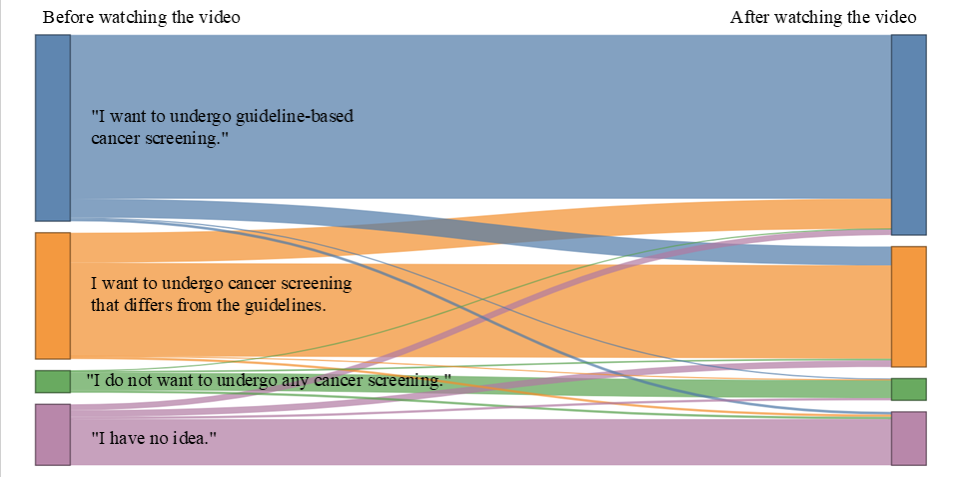
Changes in preferences regarding cancer screening before and after watching the videos. Flow widths represent the proportion of participants who were consistent in or transitioned between preference categories. Colors denote response categories and are consistent across the "before and after watching the video" nodes. Blue represents “I want to undergo guideline-based cancer screening.” Orange represents "I want to undergo cancer screening that differs from the guidelines," which included the following survey response options: “I want to undergo cancer screening at an earlier age than recommended,” “I want to undergo different types of cancer screening than recommended (eg, tumor markers or PET-CT imaging),” and “I want to undergo cancer screening at intervals shorter or longer than the recommended interval.” Green represents “I do not want to undergo any cancer screening.” Purple represents “I have no idea.”.

Figure 3 illustrates the changes in screening preferences before and after watching the video using a Sankey diagram. Among the respondents, 91 shifted toward guideline-based screening (effective group), 283 maintained non–guideline-based preferences (neutral group), and 57 transitioned from guideline-based to non–guideline-based screening preferences (counterproductive group). Table 2 presents the characteristics of these 3 groups. A history of annual colorectal cancer screening over the previous 2 years was reported to be 57.1% (52/91) in the effective group, 52.7% (149/283) in the neutral group, and 43.9% (25/57) in the counterproductive group (*P*=.04). Although no statistically significant difference was observed in median total response time, the counterproductive group tended to have a shorter response time (436 seconds) than the effective group (459 seconds) and the neutral group (464 seconds). The density distribution of the total response times for these groups is shown in Multimedia Appendix 8. Before watching the video, 383 respondents preferred non–guideline-based cancer screening, and after viewing the video, 91 (23.8%) shifted to a preference for guideline-based screening.

**Table 2:** Characteristics of respondents in the effective, neutral, and counterproductive groups (N=431).

Characteristics		Effective group (n=91)	Neutral group (n=283)	Counterproductive group (n=57)	*P* value	
Age (years), mean (SD)		50.0 (7.1)	48.5 (7.6)	48.8 (7.8)	.26	
**Age (years), n (%)**						.61
	30-39	8 (8.8)	43 (15.2)	7 (12.3)		
	40-49	31 (34.1)	85 (30.0)	17 (29.8)		
	50-60	52 (57.1)	155 (54.8)	33 (57.9)		
**Sex, n (%)**						
	Female	49 (53.8)	138 (48.8)	29 (50.9)	.70	
**Marital status, n (%)**						
	Unmarried	34 (37.4)	101 (35.7)	20 (35.1)	.95	
**Education, n (%)**						
	University degree or higher	48 (52.7)	152 (53.7)	23 (40.4)	.18	
**Income (Japanese yen; million; US $1.00 = ¥145), n (%)**						.93
	≤4.00	22 (24.2)	66 (23.3)	16 (28.1)		
	4.01-6.00	17 (18.7)	56 (19.8)	12 (21.1)		
	6.01-8.00	23 (25.3)	52 (18.4)	12 (21.1)		
	8.01-10.00	13 (14.3)	43 (15.2)	6 (10.5)		
	10.01-12.00	6 (6.6)	29 (10.2)	5 (8.8)		
	12.01-15.00	7 (7.7)	19 (6.7)	4 (7.0)		
	≥15.01	3 (3.3)	18 (6.4)	2 (3.5)		
**History of colorectal cancer screening, n (%)**						.04
	Annually (past 2 years)	52 (57.1)	149 (52.7)	25 (43.9)		
	Once (past 2 years)	12 (13.2)	15 (5.3)	4 (7.0)		
	Earlier than past 2 years	4 (4.4)	38 (13.4)	11 (19.3)		
	Never received	23 (25.3)	76 (26.9)	16 (28.1)		
	Do not know	0 (0.0)	5 (1.8)	1 (1.8)		
Total response time (seconds), median (IQR)		459 (366–603)	464 (383–600)	436 (356–522)	.22	

## Discussion

### Principal Findings

In this study, we aimed to identify effective educational strategies to convey the risk of false-positive results associated with non–guideline-based cancer screening. No statistically significant differences were observed in preferences for guideline-based cancer screening among the 3 videos designed to educate participants regarding the harm caused by false-positive results. The subjective evaluation provided additional insights into the effectiveness of video-based educational methods beyond the primary preference outcome. Among men, the evaluation of the narrative regarding breast cancer screening yielded lower scores for relevance and willingness to undergo guideline-based screening compared with evaluations among women. In contrast, the breast cancer screening narrative was generally received more favorably by women than the tumor marker narrative presented by a male character. Although not specified in the protocol beforehand, the McNemar test indicated a significant change in preferences after watching the videos. Among the 383 respondents who initially preferred non–guideline-based cancer screening, only 91 (23.8%) changed their preference to guideline-based screening after watching videos. The effective group underwent annual colorectal cancer screening. Meanwhile, the counterproductive group showed shorter response times than the effective and neutral groups.

Narrative educational videos are not expected to be more effective than logical or explanatory videos in cancer screening education. However, according to a systematic review of health education using videos [23], narrative presentations that evoke real-world situations are more effective in promoting behavioral change than the simple didactic delivery of information. Therefore, we created a control video that provided information and 2 narrative videos and hypothesized that narrative videos would be more effective than the control video; however, our results did not support this hypothesis. The findings of this study are consistent with those of a previous study examining the impact of different animation presentation formats on educational outcomes [24]. The previous study presented animated videos that explained the advantages and disadvantages of colorectal cancer screening using 3 formats: videos with animated pictographs, videos with static pictographs, and audio booklets accompanied by static pictographs. No significant differences were observed among the 3 formats in terms of knowledge acquisition or screening participation [24]. In contrast, an intervention study on lung cancer screening [25] reported that presenting an informational film in addition to a booklet was more effective than presenting only a booklet. The addition of films was found to enhance knowledge acquisition, both objectively and subjectively [25]. The absence of a statistically significant difference observed in this study may be explained by the fact that the outcome measure focused on changes in preference rather than knowledge acquisition. A systematic review of the effectiveness of animated videos found that they consistently led to greater knowledge acquisition than other educational materials and standard care; however, no clear evidence was available regarding their effectiveness in influencing attitudes and perceptions [20]. Therefore, future intervention studies are required to identify appropriate methods for presenting animated videos.

The absence of statistically significant differences in the primary outcome may also be explained in other ways. The intervention intensity may have been insufficient: all videos were brief and delivered in a single exposure, which might limit their effect on preferences. Measurement sensitivity may have been limited. The primary outcome was dichotomized into guideline-based preference vs all other options immediately after exposure, which may have masked more nuanced shifts, including the avoidance of non–guideline-based options. In addition, baseline preferences and audience heterogeneity may have contributed to the null findings. Nearly half of the respondents (565/ 1200, 47.1%) preferred guideline-based screening even before viewing the videos, which may have reduced the room for improvement (ceiling effects). Conversely, respondents with strong preexisting beliefs favoring early or intensive screening may require more tailored or repeated messaging to shift preferences. Considering these reasons, future intervention studies should be designed to examine differences in effectiveness across educational methods.

Narrative videos that focus on breast cancer may be less effective or appropriate for male audiences. The scores for relevance and willingness to undergo guideline-based screening for video B were lower among men than among women. Narrative transportation theory suggests that narrative messages are more persuasive when viewers perceive the story as personally relevant and can identify with the protagonist [19]. A breast cancer–focused narrative may have been less self-relevant for male respondents, potentially reducing narrative engagement and transportation and thereby explaining the lower ratings for perceived relevance and willingness to undergo guideline-based screening observed among men for video B. Audience engagement has been identified as a key factor even in the effectiveness of educational videos [26]. Because breast cancer is typically not viewed as a personal health issue by men, it may be less effective in fostering engagement among men. This interpretation is further supported by the finding that women tended to rate the breast cancer video more favorably than video C, which showed a male undergoing multiple tumor marker tests.

The harmful effects of screening are often regarded by screening participants as important prior knowledge when making informed decisions. A qualitative study in Australia [14] used focus group discussions on harms regarding mammography and reported that several participants considered this information important for making informed choices and expressed a strong desire to be informed in advance. However, a randomized controlled trial conducted in Australia [27] showed that informing participants about the harm of breast cancer screening did not reduce their willingness to undergo screening and helped maintain their level of knowledge. Although providing balanced information is important, public information sources often fail to adequately address the associated risks [28]. In this regard, video-based education offers repeatability and accessibility across geographic boundaries [29]. Accordingly, video B in this study helps resolve this issue for women.

A before-and-after comparison of video-based education suggests a potential influence on participants’ intentions. Although the videos were only 100 seconds long, an increase in the preference for guideline-based cancer screening was observed. Individuals who undergo annual colorectal cancer screening are generally more likely to actively participate in screening for other cancers. If such individuals become more inclined to follow guideline-based screening after learning about false-positive results, it may indicate that the educational videos were effectively communicated to an appropriate audience.

Notably, education regarding the risks of non–guideline-based screening had a limited impact on changing individuals’ preferences for guideline-based cancer screening. In this study, fewer than 1 in 4 respondents changed their preference from non–guideline-based to guideline-based screening after watching the videos. This may be partly explained by the misleading positive feedback loops that are intrinsic to cancer screening [30]. When cancer is detected through screening, the benefit of early detection tends to be emphasized, whereas a negative result reinforces the perception that one is responsible for one’s health. Moreover, the cost and pain associated with follow-up examinations are often perceived as acceptable trade-offs for survival [30]. In addition, negative feedback from personal screening experiences is rare, which may help sustain positive perceptions. From a physician’s perspective, considering the seriousness of identifying prostate cancer after not undergoing screening, the decision to undergo screening is a no-loss choice compared with not undergoing screening [31]. Furthermore, specific harms, such as fear, pain, and financial burden, are prioritized by physicians to explain the harms of screening. Consequently, problem-based learning curricula aimed at helping medical students understand harm are being developed and evaluated for effectiveness to address this unconscious bias [32].

This study contributes to the literature on cancer screening education in certain ways. First, although many educational video interventions primarily aim to increase screening participation, our videos specifically communicated the potential harms of non–guideline-based screening and evaluated changes in preference for guideline-based screening. Second, we compared 3 brief videos in online delivery settings and assessed multiple perceptions that can guide the development of video-based educational materials. Third, observed sex differences in perceived relevance for the breast cancer narrative suggest that matching content to the target audience is important. Fourth, considering the trend toward earlier and excessive cancer screening in Japan, the finding that a large number of participants still preferred screening beyond recommended levels even after viewing the educational materials is an important insight. Future efforts should focus on clarifying the underlying problems and exploring effective intervention methods.

### Limitations

Despite the positive outcomes, this study has some limitations. First, the generalizability of the results may be limited. The respondents in this study were individuals who were able to spend at least 5 minutes completing the online survey. Therefore, the findings may not be generalizable to populations that are less inclined to participate in online surveys. Second, the primary outcome was a self-reported preference assessed immediately after video exposure, which may not translate into actual screening behaviors in real-world settings. This limits the external validity and practical applicability of our findings. Future studies should incorporate longer follow-up periods and objective end points, such as medical records from hospitals and clinics, to evaluate whether brief video-based education results in behavioral change. Third, the circumstances under which respondents watched the educational videos during the survey, likely influencing their engagement and responses, are unclear. At the beginning of the survey, the respondents were instructed to watch the video until the end while listening to the audio; however, we were unable to verify whether they followed these instructions. For example, if the participants joined the survey while on a train without using headphones, they may have completed it without listening to the audio. Fourth, the before-and-after analysis was exploratory and not prespecified in the trial protocol. Although this analysis provides insight into immediate within-person shifts after exposure, it may be susceptible to analytic flexibility and should be interpreted cautiously. Therefore, the primary inference of this study is based on the prespecified between-group comparison of video formats.

### Conclusions

In this online pseudorandomized trial, narrative videos did not demonstrate a greater effect than explanatory videos in increasing preferences for guideline-based cancer screening. This negative finding suggests that concise, single-video interventions are unlikely to produce large format-specific effects and that preferences for excessive screening may persist despite education about the harms of false-positive results. To address beliefs supporting screening beyond recommended levels, future interventions should consider increasing intensity and tailoring education content to individual characteristics.

## Appendix

Multimedia Appendix 1 CONSORT 2025 checklist.

Multimedia Appendix 2 Structures of 3 types of cancer screening promotion videos.

Multimedia Appendix 3 Video A. Explanatory video on guideline-based cancer screening and false positives (84 seconds). The presenter uses a whiteboard and figures to describe the screening pathway from screening to confirmatory testing and to explain why false positives are more common when cancer prevalence is low. The video summarizes potential harm, such as anxiety, time, and costs of follow-up testing, alongside benefits of early detection and concludes with the message to follow evidence-based screening guidelines.

Multimedia Appendix 4 Video B. Narrative video showing a false-positive experience after early-age breast cancer screening (77 seconds). A female employee receives an abnormal result, struggles to arrange follow-up, and experiences stress and practical burdens, such as repeated visits and time off work, before learning that no cancer is present. The video highlights that breast cancer risk is low at age 30 and ends by recommending starting the screening at the recommended age.

Multimedia Appendix 5 Video C. Narrative video depicting false-positive results after tumor-marker testing (77 seconds). A male employee undergoes tumor-marker testing, receives abnormal results, and undergoes multiple follow-up examinations over several months, resulting in no abnormality. The video emphasizes the harms of unnecessary testing, such as monetary costs and repeated radiation exposure, and recommends to choose guideline-based cancer screening.

Multimedia Appendix 6 Complete results from the analysis for the primary outcome.

Multimedia Appendix 7 All results from the 7-item evaluation with mean score with 95% CI for each video.

Multimedia Appendix 8 Density distribution of total response time in 3 change groups.

## Abbreviations

aOR: adjusted odds ratio
PET-CT: positron emission tomography-computed tomography

## Data Availability

Data sharing is not applicable to this article as no data sets were generated or analyzed during this study. Reasonable requests for additional information regarding the study can be directed to the corresponding author.

## References

[1] Crosby D, Bhatia S, Brindle KM, Coussens LM, Dive C, Emberton M, Esener S, Fitzgerald RC, Gambhir SS, Kuhn P, Rebbeck TR, Balasubramanian S (2022). Early detection of cancer. Science.

[2] Wright K, Shkabari B, Booth C, Knopf K, Tregear M, Gyawali B (2025). Congruence of cancer screening recommendations between the USPSTF and the top ten US cancer centers: a cross sectional study. EClinicalMedicine.

[3] Hamashima C (2018). Cancer screening guidelines and policy making: 15 years of experience in cancer screening guideline development in Japan. Jpn J Clin Oncol.

[4] Leavy MB, Starzyk K, Myers E, Curhan G, Gliklich R (2020). Using real-world evidence to support a changing paradigm for cancer screening: a commentary. Pharmacoepidemiol Drug Saf.

[5] Ghotbi N, Iwanaga M, Ohtsuru A, Ogawa Y, Yamashita S (2007). Cancer screening with whole-body PET/CT for healthy asymptomatic people in Japan: re-evaluation of its test validity and radiation exposure. Asian Pac J Cancer Prev.

[6] Rintala S, Dahlstrom KR, Franco EL, Louvanto K (2023). A synthesis of evidence for cancer-specific screening interventions: a preventive medicine golden jubilee review. Prev Med.

[7] Rasmussen JF, Siersma V, Malmqvist J, Brodersen J (2020). Psychosocial consequences of false positives in the Danish Lung Cancer CT Screening Trial: a nested matched cohort study. BMJ Open.

[8] Croswell JM, Kramer BS, Kreimer AR, Prorok PC, Xu J, Baker SG, Fagerstrom R, Riley TL, Clapp JD, Berg CD, Gohagan JK, Andriole GL, Chia D, Church TR, Crawford ED, Fouad MN, Gelmann EP, Lamerato L, Reding DJ, Schoen RE (2009). Cumulative incidence of false-positive results in repeated, multimodal cancer screening. Ann Fam Med.

[9] Kasahara Y, Kawai M, Tsuji I, Tohno E, Yokoe T, Irahara M, Tangoku A, Ohuchi N (2013). Harms of screening mammography for breast cancer in Japanese women. Breast Cancer.

[10] Tsunematsu M, Kakehashi M (2015). An analysis of mass screening strategies using a mathematical model: comparison of breast cancer screening in Japan and the United States. J Epidemiol.

[11] Ford ME, Havstad SL, Flickinger L, Johnson CC (2003). Examining the effects of false positive lung cancer screening results on subsequent lung cancer screening adherence. Cancer Epidemiol Biomarkers Prev.

[12] Miglioretti DL, Abraham L, Sprague BL, Lee CI, Bissell MC, Ho TH, Bowles EJ, Henderson LM, Hubbard RA, Tosteson AN, Kerlikowske K (2024). Association between false-positive results and return to screening mammography in the breast cancer surveillance consortium cohort. Ann Intern Med.

[13] Klabunde CN, Vernon SW, Nadel MR, Breen N, Seeff LC, Brown ML (2005). Barriers to colorectal cancer screening: a comparison of reports from primary care physicians and average-risk adults. Med Care.

[14] Hersch J, Jansen J, Barratt A, Irwig L, Houssami N, Howard K, Dhillon H, McCaffery K (2013). Women's views on overdiagnosis in breast cancer screening: a qualitative study. BMJ.

[15] Noman S, Shahar HK, Abdul Rahman H, Ismail S, Abdulwahid Al-Jaberi M, Azzani M (2020). The effectiveness of educational interventions on breast cancer screening uptake, knowledge, and beliefs among women: a systematic review. Int J Environ Res Public Health.

[16] Wu S, Chalela P, Ramirez AG (2023). Changes in knowledge and awareness for a community-based cancer screening educational program. Arch Public Health.

[17] Minamitani M, Tatemichi M, Mukai T, Katano A, Ohira S, Nakagawa K (2024). Adherence to national guidelines for colorectal, breast, and cervical cancer screenings in Japanese workplaces: a survey-based classification of enterprises' practices into "overscreening," "underscreening," and "guideline-adherence screening". BMC Public Health.

[18] Minamimoto R, Senda M, Jinnouchi S, Terauchi T, Yoshida T, Murano T, Fukuda H, Iinuma T, Uno K, Nishizawa S, Tsukamoto E, Iwata H, Inoue T, Oguchi K, Nakashima R, Inoue T (2013). The current status of an FDG-PET cancer screening program in Japan, based on a 4-year (2006-2009) nationwide survey. Ann Nucl Med.

[19] Green MC, Fitzgerald K, Nussbaum JF (2017). Transportation theory applied to health and risk messaging. Oxford Research Encyclopedia of Communication.

[20] Moe-Byrne T, Evans E, Benhebil N, Knapp P (2022). The effectiveness of video animations as information tools for patients and the general public: a systematic review. Front Digit Health.

[21] Lau J, Lim TZ, Jianlin Wong G, Tan KK (2020). The health belief model and colorectal cancer screening in the general population: a systematic review. Prev Med Rep.

[22] Correia D, Kokole D, Rehm J, Tran A, Ferreira-Borges C, Galea G, Likki T, Olsen A, Neufeld M (2024). Effect of alcohol health warning labels on knowledge related to the ill effects of alcohol on cancer risk and their public perceptions in 14 European countries: an online survey experiment. Lancet Public Health.

[23] Abu Abed M, Himmel W, Vormfelde S, Koschack J (2014). Video-assisted patient education to modify behavior: a systematic review. Patient Educ Couns.

[24] Housten AJ, Kamath GR, Bevers TB, Cantor SB, Dixon N, Hite A, Kallen MA, Leal VB, Li L, Volk RJ (2020). Does animation improve comprehension of risk information in patients with low health literacy? A randomized trial. Med Decis Making.

[25] Ruparel M, Quaife SL, Ghimire B, Dickson JL, Bhowmik A, Navani N, Baldwin DR, Duffy S, Waller J, Janes SM (2019). Impact of a lung cancer screening information film on informed decision-making: a randomized trial. Ann Am Thorac Soc.

[26] Brame CJ (2016). Effective educational videos: principles and guidelines for maximizing student learning from video content. CBE Life Sci Educ.

[27] Hersch J, Barratt A, McGeechan K, Jansen J, Houssami N, Dhillon H, Jacklyn G, Irwig L, McCaffery K (2021). Informing women about overdetection in breast cancer screening: two-year outcomes from a randomized trial. J Natl Cancer Inst.

[28] Attena F, Cancellieri M, Pelullo CP (2016). Scarce information about breast cancer screening: an Italian websites analysis. Medicine (Baltimore).

[29] Chatterjee A, Strong G, Meinert E, Milne-Ives M, Halkes M, Wyatt-Haines E (2021). The use of video for patient information and education: a scoping review of the variability and effectiveness of interventions. Patient Educ Couns.

[30] Kramer BS, Croswell JM (2009). Cancer screening: the clash of science and intuition. Annu Rev Med.

[31] Ransohoff DF, McNaughton Collins M, Fowler FJ (2002). Why is prostate cancer screening so common when the evidence is so uncertain? A system without negative feedback. Am J Med.

[32] Ekhlas S, Lang E, Dickinson J (2023). Optimizing overdiagnosis education for medical students. BMJ Evid Based Med.

[33] GPT-5.2. OpenAI.

