# Supplementary material for: Effectiveness of Educational Videos in Encouraging Preferences for Guideline-Based Cancer Screening in Japan: Three-Arm Pseudorandomized Controlled Trial
Source: J Med Internet Res. 2026 Feb 12;28:e82322. doi: 10.2196/82322 (PMC12946783; doi:10.2196/82322)
Supplement: Multimedia Appendix 2 [file jmir_v28i1e82322_app2.docx]

# Multimedia Appendix 2. Structures of three types of cancer screening promotion videos

|  | Video A | Video B | Video C |
| --- | --- | --- | --- |
| Overview | | | |
| Style | Explanatory format | Narrative format | Narrative format |
| Summary | Potential drawbacks including false-positive risks of excessive cancer screening were explained by visual aids such as graphs and quantitative data | The narrative story showed a woman in her 30s who was ultimately found to have a false-positive result after a breast-cancer screening. | The narrative story showed a case of a working man who underwent multiple tumor marker tests, received several positive results, and consequently had to undergo extensive follow-up testing. |
| Key  messages | “Follow the guidelines for cancer screenings.” | | |
|  | Cancer screening has not only benefits but also the risk of false positives. | Undergoing cancer screening before the recommended age carries the risk of false positives. | Undergoing tests not included in recommended cancer screening programs carries the risk of false positives. |
| Narrative Transportation Theory | | | |
| Protagonist & identification | Woman in a white coat  Characters with whom viewers can easily identify do not appear. | Female employee  Breast cancer screening targets become easier to identify. | Male employee  Participants who have considered tumor marker testing become easier to identify. |
| Personal relevance cues | Not applicable | Since the target audience consists of workers, we added a scene to the video showing the results of a workplace health checkup being notified. | Since the target audience consists of workers, we added a scene to the video depicting a workplace conversation about health checkups. |
| Emotional valence | Not applicable  (The benefits and harms of cancer screening are explained without emotional bias.) | Anxiety, financial burden, and time commitment resulting from false positive | Anxiety, financial burden, and time commitment resulting from false positive |
| Transportation supports | Not applicable  (Explain using a whiteboard) | Progressing at a brisk pace with short cuts  Using narration and background music | Progressing at a brisk pace with short cuts  Using narration and background music |
| Counterarguing  reduction  strategy | Not applicable | Balanced tone; autonomy-supportive; non-judgmental narrative | Balanced tone; autonomy-supportive; non-judgmental narrative |
| Others | | | |
| Focused  cancer | Stomach cancer | Breast cancer | Stomach, colon, and lung cancer |
| Duration | 84 seconds | 77 seconds | 77 seconds |
| Visual  style | 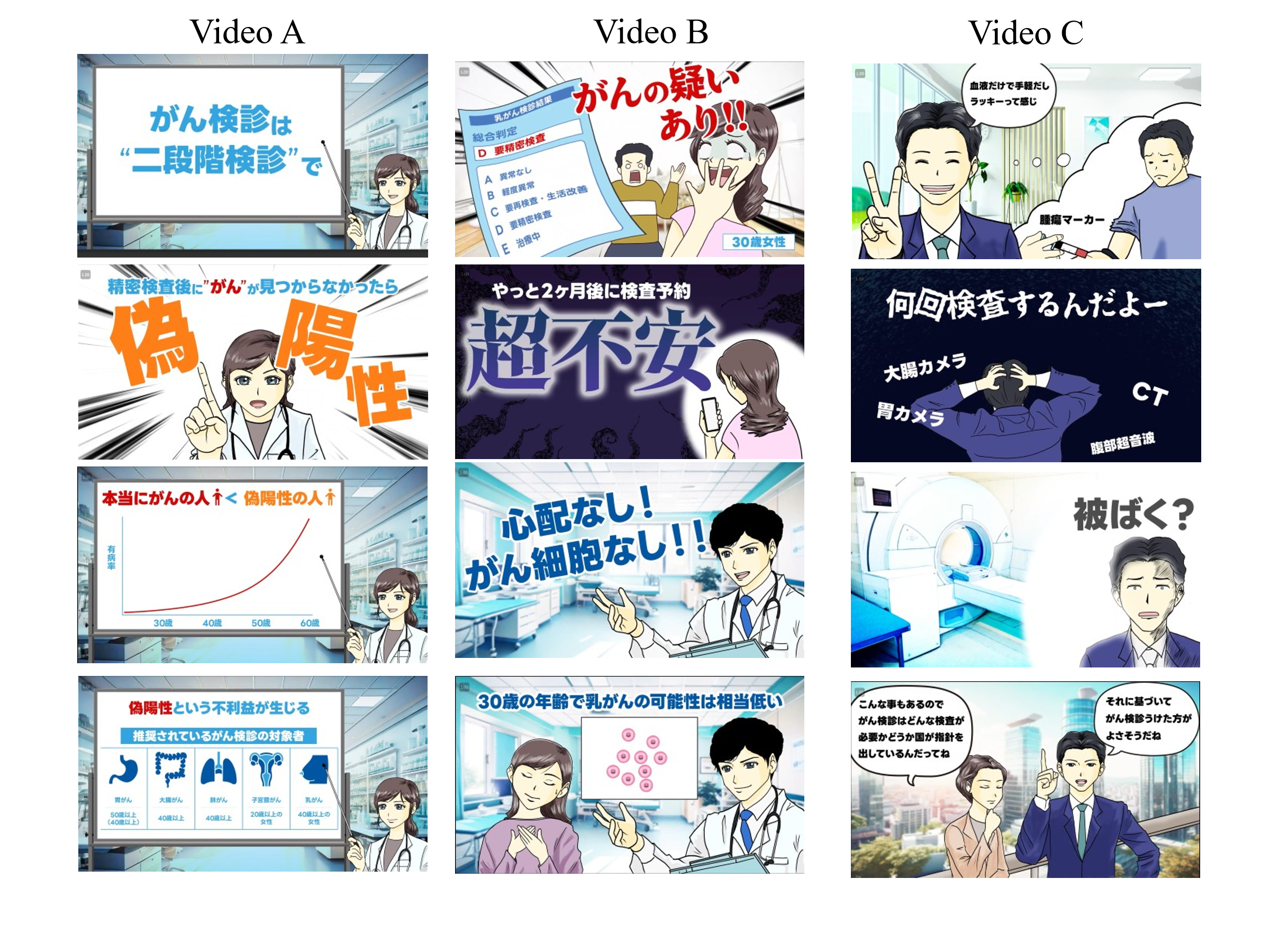 | 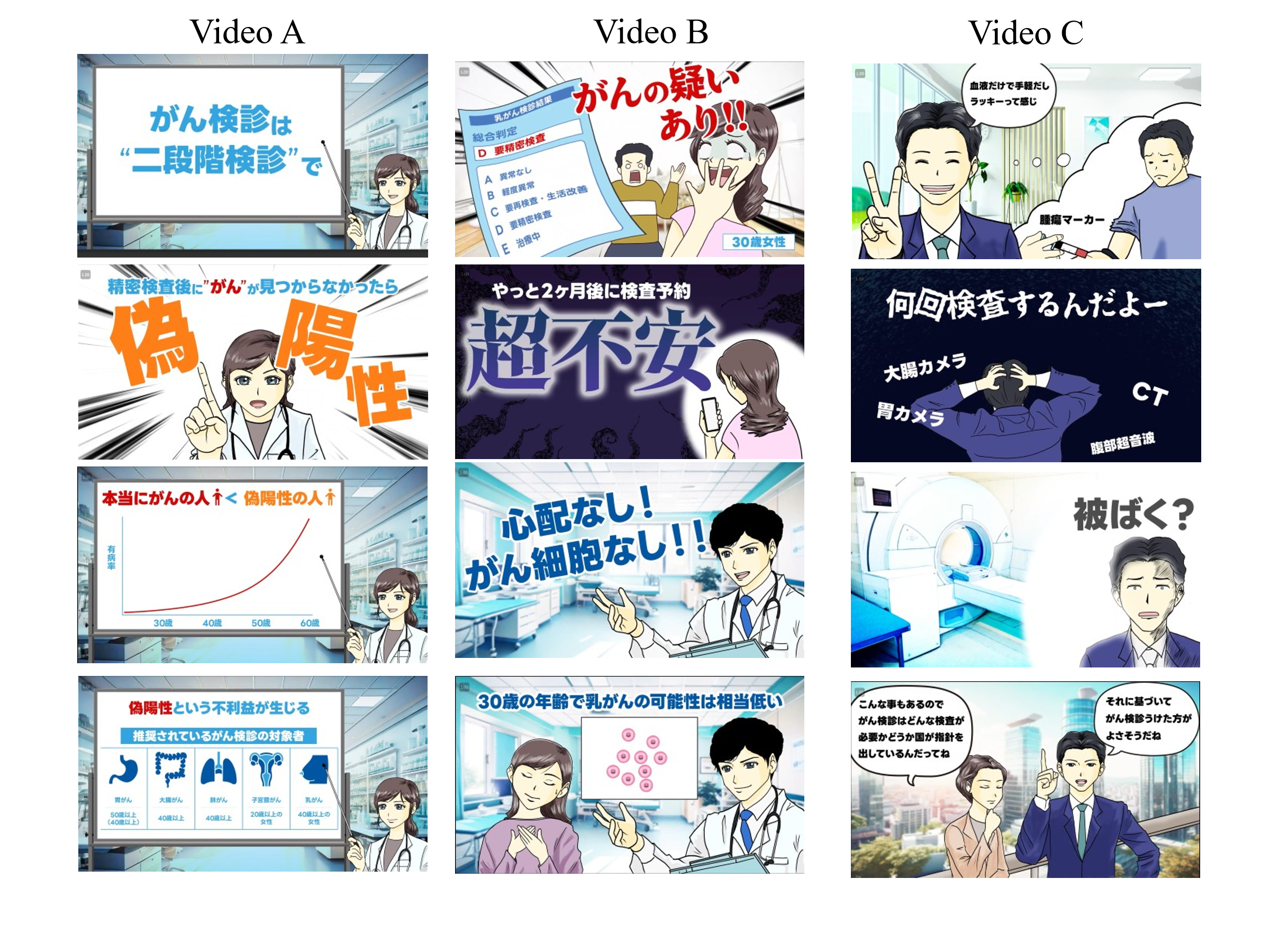 | 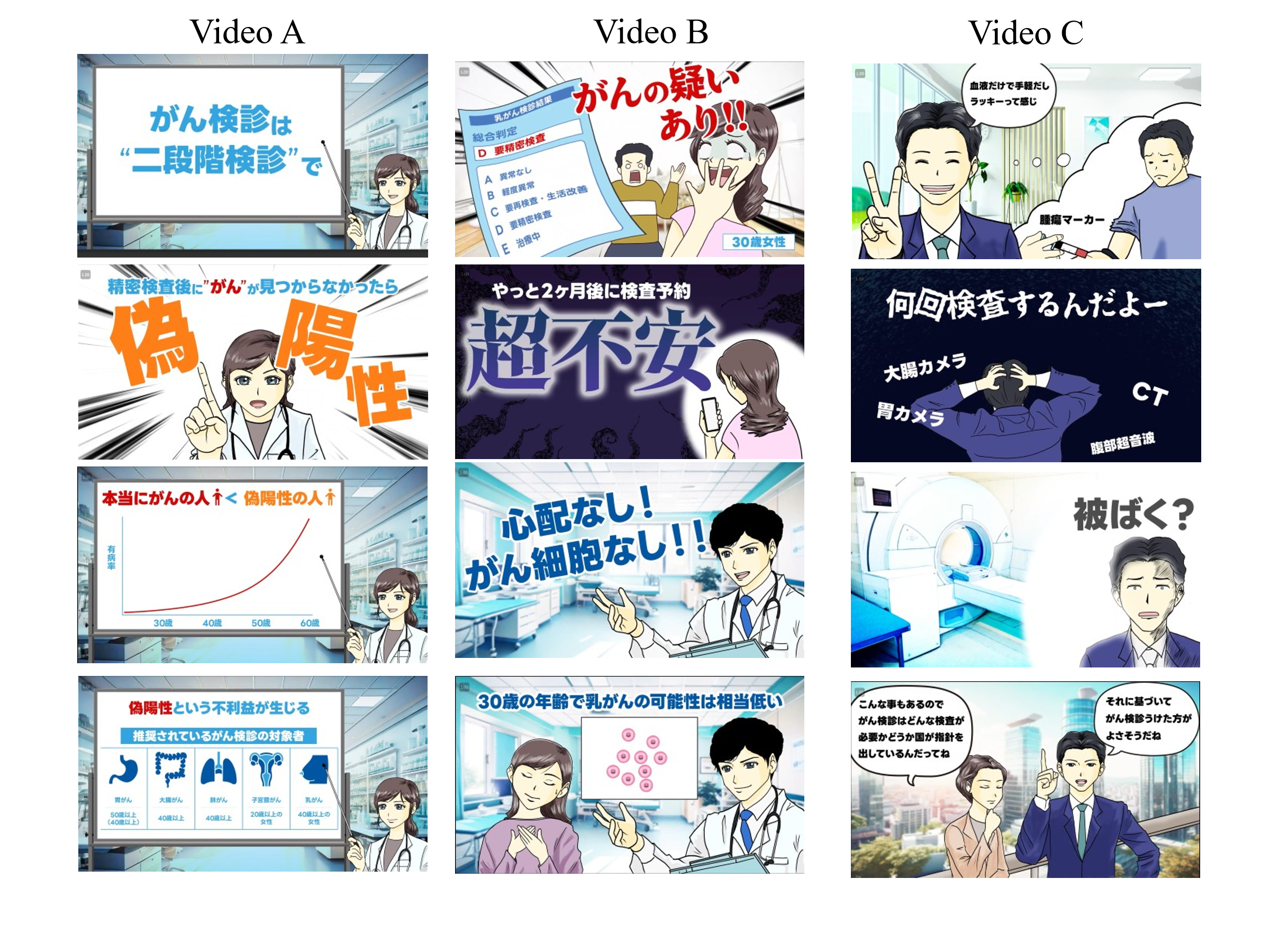 |
| Timing  &  Contents | (00:01) Cancer screening is described as a two-step process: an initial screening test narrows down who “might have cancer,” followed by confirmatory (detailed) testing.  (00:13) If the detailed exam finds no cancer after a positive/abnormal screening result, that outcome is called a false positive.  (00:25) Main drivers of false positives are test accuracy and disease prevalence in the screened population.  (00:29) Example given: for 30-year-olds, stomach cancer prevalence is extremely low (illustrated as about 1 in 50,000), and thus, even with a positive screening result, the chance of truly having cancer is low, meaning false positives become more common.  (00:41) Being told “cancer is suspected” can cause psychological distress, and the follow-up process (booking, attending appointments, receiving explanations) creates time and financial burdens owing to repeated hospital visits.  (01:00) Screening also has clear benefits: it can detect cancer early, allow less invasive treatment, and save lives.  (01:12) However, getting screened at an earlier age than recommended by guidelines can increase harms such as false positives.  (01:21) The takeaway message: follow evidence-based screening guidelines rather than starting screening earlier than recommended. | (00:01) After a workplace health check labels her as “needs further examination,” she rushes to book follow-up tests but the earliest appointment is two months later, prolonging anxiety.  (00:10) Repeated follow-up visits require taking paid leave multiple times and lead to substantial out-of-pocket costs, emphasizing psychological, time, and financial burdens.  (00:27) On the day of the results, the doctor tells her no malignant cells were found, and she feels relieved.  (00:35) The doctor explains that at the age of 30 years, the likelihood of breast cancer is very low, highlighting how worry can be amplified in younger adults.  (00:42) The video notes that in younger women, imaging may show white shadows more easily, which can trigger abnormal findings despite no true disease, such as false positives, as well as misleading findings.  (00:53) She questions whether breast cancer screening at the age of 30 years was necessary, given the heavy burden and stress.  (01:07) The takeaway: the government recommends breast cancer screening starting at the age of 40 years or later, urging viewers to follow guideline-based screening. | (00:01) A worker takes a company-subsidized health check and feels lucky because it’s just a quick blood test.  (00:13) The results later show abnormalities in several tumor-marker items, causing worry.  (00:26) The worker is advised to undergo multiple follow-up tests (e.g., stomach and colorectal exams, plus lung testing) and feels the burden: “How many tests do I need?”  (00:36) After further evaluation, there is no significant abnormality except a small shadow in the lung, suggesting a false positive overall.  (00:46) Over about 3 months, they undergo many tests and have to take time off work, describing the experience as stressful and disruptive.  (00:55) A repeat CT in 6 months is planned, raising concerns about radiation exposure.  (01:04) The takeaway: because situations like this can happen, the government provides screening guidelines, and it’s safer to choose guideline-based cancer screening. |
