# Supplementary material for: Effectiveness of Educational Videos in Encouraging Preferences for Guideline-Based Cancer Screening in Japan: Three-Arm Pseudorandomized Controlled Trial
Source: J Med Internet Res. 2026 Feb 12;28:e82322. doi: 10.2196/82322 (PMC12946783; doi:10.2196/82322)
Supplement: Multimedia Appendix 6 [file jmir_v28i1e82322_app6.docx]

# Multimedia Appendix 6. Complete results from the analysis for primary outcome

|  |  | aOR* | 95% CI† | | |
| --- | --- | --- | --- | --- | --- |
| Video | |  |  |  |  |
|  | A | 1.00 |  |  |  |
|  | B | 0.89 | 0.59 | – | 1.32 |
|  | C | 0.98 | 0.65 | – | 1.46 |
| Sex | |  |  |  |  |
|  | Men | 1.00 |  |  |  |
|  | Women | 1.50 | 1.06 | – | 2.14 |
| Age | | 1.03 | 1.00 | – | 1.05 |
| Education | |  |  |  |  |
|  | Less than university | 1.00 |  |  |  |
|  | university or more | 1.52 | 1.08 | – | 2.14 |
| Preference for cancer screening before watching the video | |  |  |  |  |
|  | “I want to undergo cancer screening in accordance with the guidelines.” | 1.00 |  |  |  |
|  | I want to undergo cancer screening that differs from the guidelines. | 0.04 | 0.03 | – | 0.06 |
|  | “I don't want to get screened for cancer.” | 0.00 | 0.00 | – | 0.01 |
|  | “I have no idea.” | 0.01 | 0.01 | – | 0.03 |

*aOR: adjusted odds ratio

†CI: confidence intervals
