# Supplementary material for: Effectiveness of Educational Videos in Encouraging Preferences for Guideline-Based Cancer Screening in Japan: Three-Arm Pseudorandomized Controlled Trial
Source: J Med Internet Res. 2026 Feb 12;28:e82322. doi: 10.2196/82322 (PMC12946783; doi:10.2196/82322)
Supplement: Multimedia Appendix 7 [file jmir_v28i1e82322_app7.docx]

# Multimedia Appendix 7. All results from the 7-item evaluation with mean score with 95% CI for each video.

| Video_Sex | A_Men | A_Women | B_Men | B_Women | C_Men | C_Women |
| --- | --- | --- | --- | --- | --- | --- |
| Relevance | 3.51 (3.38–3.64) | 3.48 (3.35–3.60) | 2.55 (2.39–2.71) | 3.54 (3.40–3.69) | 3.40 (3.27–3.53) | 3.31 (3.17–3.45) |
| Clarity | 3.87 (3.75–3.99) | 3.88 (3.77–3.99) | 3.80 (3.67–3.93) | 3.98 (3.87–4.09) | 3.85 (3.74–3.97) | 3.90 (3.78–4.01) |
| Informativeness | 3.52 (3.41–3.64) | 3.52 (3.41–3.63) | 3.36 (3.23–3.49) | 3.58 (3.46–3.70) | 3.45 (3.33–3.57) | 3.43 (3.30–3.56) |
| Acceptability | 3.75 (3.63–3.87) | 3.71 (3.61–3.82) | 3.56 (3.44–3.69) | 3.79 (3.67–3.90) | 3.70 (3.58–3.82) | 3.73 (3.61–3.85) |
| Aversion* | 3.56 (3.43–3.69) | 3.46 (3.33–3.58) | 3.38 (3.23–3.52) | 3.52 (3.39–3.65) | 3.50 (3.38–3.63) | 3.49 (3.36–3.62) |
| Discussion | 3.06 (2.94–3.19) | 2.98 (2.87–3.10) | 2.90 (2.77–3.03) | 2.92 (2.77–3.06) | 2.96 (2.83–3.08) | 2.93 (2.79–3.07) |
| Willingness to guideline-based screening | 3.59 (3.46–3.72) | 3.68 (3.57–3.80) | 3.28 (3.14–3.42) | 3.66 (3.53–3.79) | 3.52 (3.40–3.64) | 3.48 (3.35–3.62) |

*Reversed score
