# Supplementary figures and images for: Effectiveness of Educational Videos in Encouraging Preferences for Guideline-Based Cancer Screening in Japan: Three-Arm Pseudorandomized Controlled Trial
Source: J Med Internet Res. 2026 Feb 12;28:e82322. doi: 10.2196/82322 (PMC12946783; doi:10.2196/82322)

# Multimedia Appendix 8. Density distribution of total response time in 3 change groups


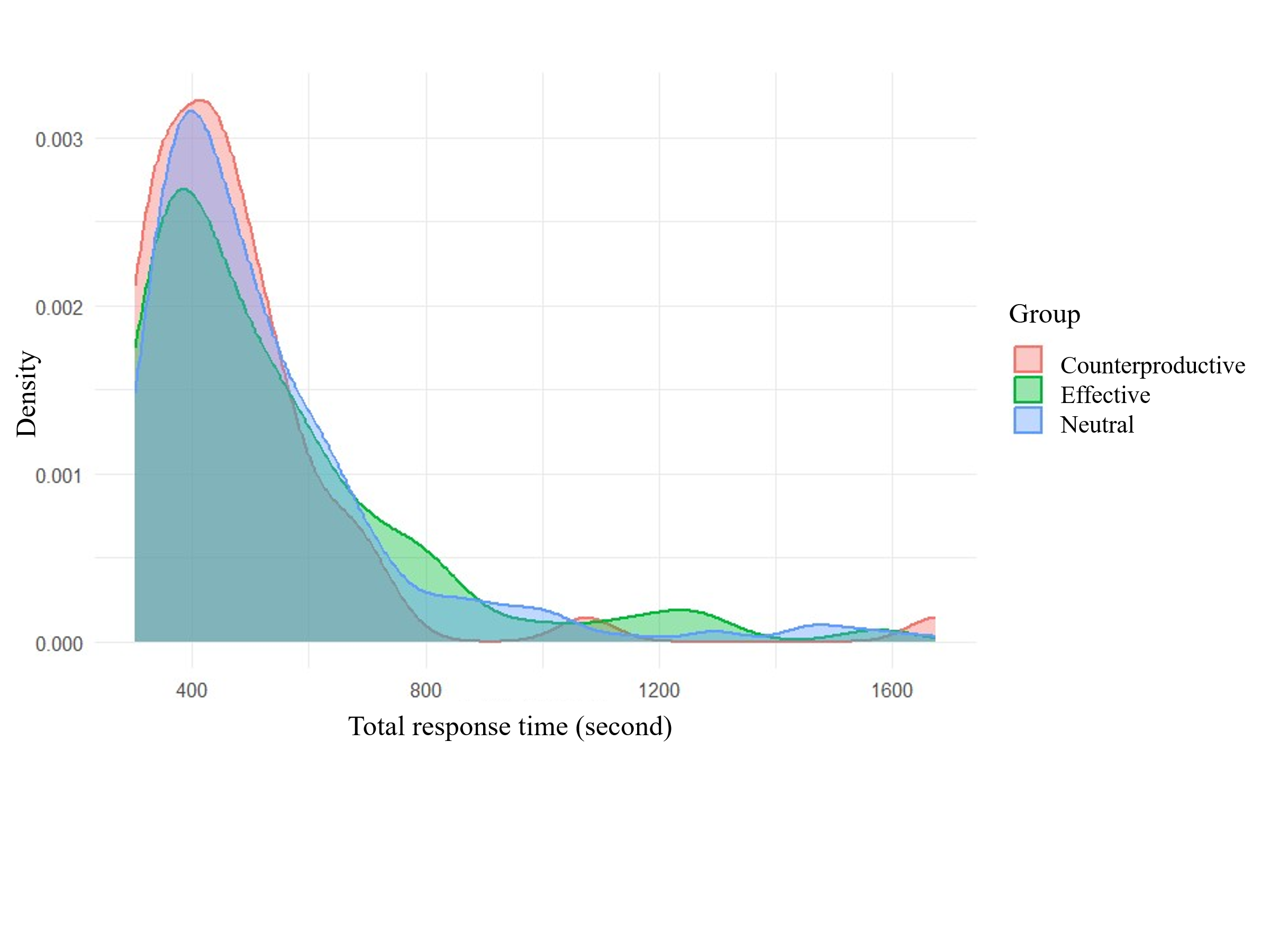

Supplement: Multimedia Appendix 8 [file jmir_v28i1e82322_app8.docx]
